# Protocols for collecting mouse PDL cells and bone marrow cells, differentiation, and data analysis

**DOI:** 10.1016/j.xpro.2024.103162

**Published:** 2024-06-26

**Authors:** Kridtapat Sirisereephap, Meircurius Dwi Condro Surboyo, Andrea L. Rosenkranz, Yutaka Terao, Koichi Tabeta, Takeyasu Maeda, George Hajishengallis, Tomoki Maekawa

**Affiliations:** 1 Faculty of Dentistry, Chulalongkorn University, Bangkok 10330, Thailand; 2 Center for Advanced Oral Science, Graduate School of Medical and Dental Sciences, Niigata University, Niigata 951-8514, Japan; 3 Faculty of Dentistry, Universitas Airlangga, Surabaya 60132, Indonesia; 4 Division of Microbiology and Infectious Diseases, Graduate School of Medical and Dental Sciences, Niigata University, Niigata 951-8514, Japan; 5 Division of Periodontology, Graduate School of Medical and Dental Sciences, Niigata University, Niigata 951-8514, Japan; 6 Department of Basic and Translational Sciences, Laboratory of Innate Immunity and Inflammation, School of Dental Medicine, University of Pennsylvania, Philadelphia, PA 19104, USA

**Keywords:** Cell Biology, Cell culture, Immunology, Model Organisms, Molecular Biology

## Abstract

Periodontal ligament cells (PDLCs) and macrophages in bone marrow cells have been widely used to investigate novel therapeutic agents to treat periodontitis. Here, we present a protocol for collecting primary mouse PDLCs and bone marrow cells. We detail steps for culturing and differentiation for both cell types and review data analysis for *in vitro* experiments using primary PDLCs and bone marrow cells. This protocol can be used to explore the impact of novel therapeutic agents using *in vitro* experiments.

For complete details on the use and execution of this protocol, please refer to Sirisereephap et al.^1^

## Highlights

• Steps for isolating and growing primary mouse PDLCs and BMMs
• Guidance on differentiating PDLCs and BMMs into osteoblasts and osteoclasts
• Data analysis steps to confirm expression of bone-related enzymatic activity

## Abstract (editor highlights)

Publisher’s note: Undertaking any experimental protocol requires adherence to local institutional guidelines for laboratory safety and ethics.

## Graphical abstract

**Figure:**
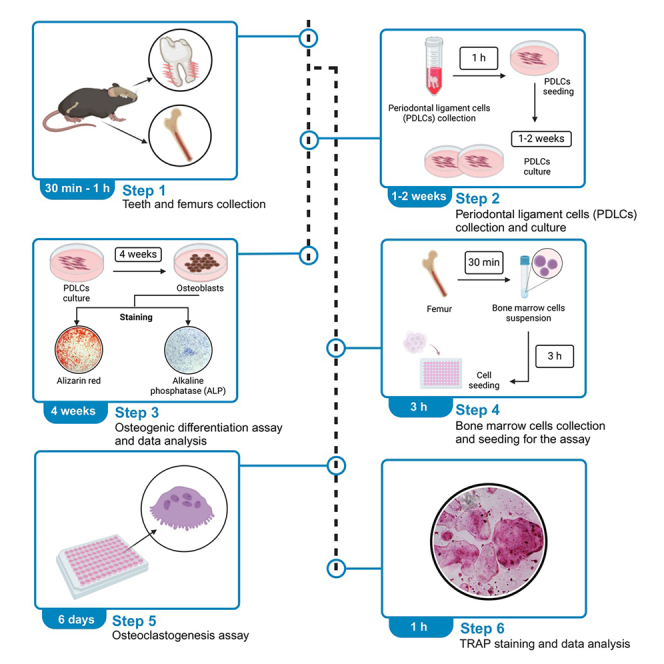

## Before you begin

The current study showing great potential to find the treatment in periodontitis is the exploration of regenerative medicine, or therapeutic agents having the capacity to promote osteogenic differentiation, to promote bone growth in a bone defect area. This involves utilizing stem cells or PDL cells to investigate how drugs can stimulate the differentiation of those cells into cells at the later stage of development, such as osteoblasts that are responsible for new bone formation.^2^

Osteogenic differentiation is the transformation of undifferentiated cells, such as mesenchymal stem cells and PDLCs, into osteoblasts, which plays a pivotal role in bone formation. PDLCs, cells derived from the periodontium that encompasses and supports the teeth, have the ability to undergo osteogenic differentiation when exposed to specific conditions.^3^ This process typically involves subjecting PDLCs to an osteogenic medium containing components such as dexamethasone, β-glycerophosphate, and ascorbic acid. These components replicate the biochemical environment necessary for the development of osteoblasts. Throughout the osteogenic differentiation process, PDLCs adopt osteoblastic characteristics, including the synthesis of extracellular matrix proteins, such as collagen, and the formation of mineralized nodules.^4^

In addition to investigating therapeutic drugs for their impact on osteogenic differentiation, the examination the drugs effect on osteoclasts offers an avenue to explore the inhibitory effects on osteoclastogenesis, thereby impeding bone loss during the progression of periodontitis.^5^

Osteoclastogenesis refers to the formation of osteoclasts, cells responsible for bone resorption. In *in vitro* studies focusing on osteoclastogenesis, the primary goal is usually to understand the factors and mechanisms influencing the differentiation and maturation of osteoclasts. Anticipated outcomes may differ based on distinct experimental conditions, cell types used, and the study objectives.^6^

Approval to work with animals must be obtained from the appropriate authorities and the institution where the work will take place. Additionally, proper training in animal handling is mandatory.

### Institutional permissions

Experiments received ethical approval from the Institutional Animal Care and Use Committee (IACUC) of Niigata University (approval no. SA00960).

### Animal housing

**Timing: 2 weeks**

1. Obtain C57BL/6 wild-type mice aged between 6 to 8 weeks and allow a 2-week acclimation period to the experimental environment. Both genders are suitable for subsequent experiments; however, considering the potential impact of sex on the immune response, it is advisable to consistently employ sex-matched control groups or use mice of the same sex for all experiments.

**CRITICAL:** Ensure that experimental groups are matched in terms of both sex and age, as these variables can have an impact on the results. Additionally, when working with genetically modified mice, employ wild-type littermates as control groups to mitigate the influence of diverse genetic backgrounds as a potential explanation for observed differences between experimental groups.

### Preparation for collecting primary mouse periodontal ligament cells (PDLCs)

**Timing: 2 h (for step 2)**

**Timing: 1 h (for steps 3–6)**

The day prior to the scheduled mouse sacrifice

**Figure 1:**
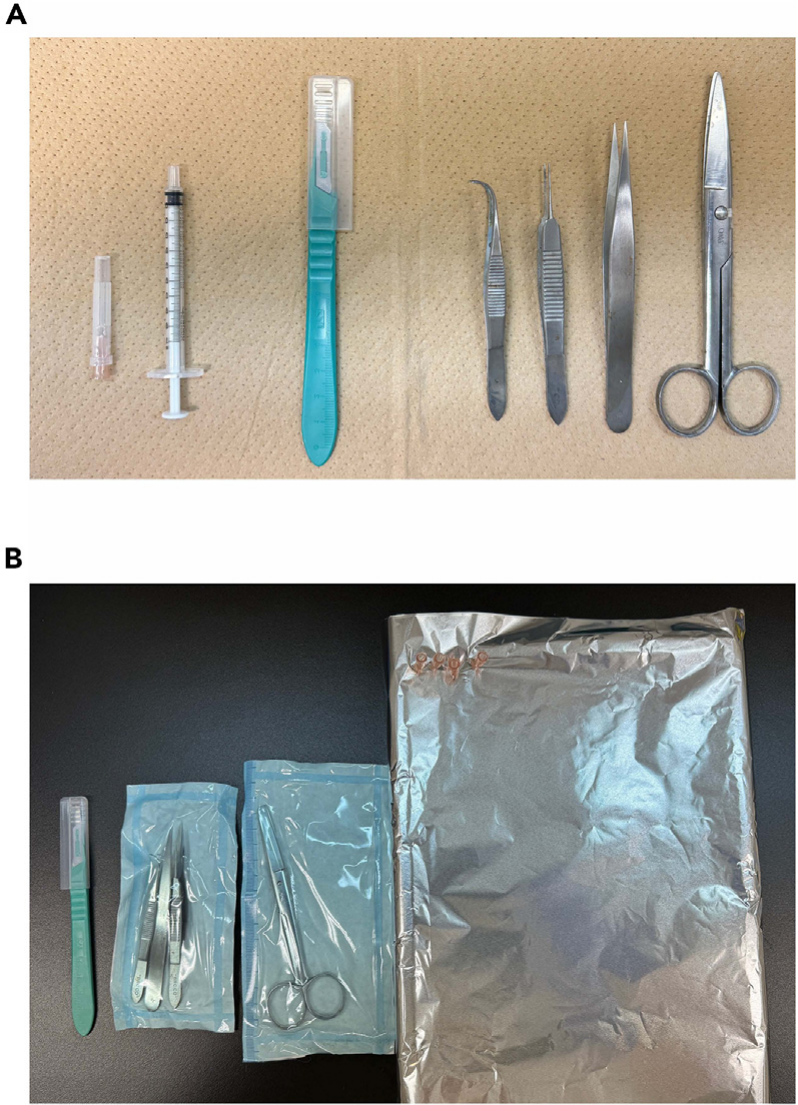
Tools for collecting PDLCs and primary bone marrow cells from mouse (A) From left to right: 26G needle, 1 mL syringe, no. 10 scalpel blade, curved forceps (Perry), suture-tying forceps, Dumont forceps, and surgical scissors. (B) The actual setup for sample collection. The styrene foam is covered with aluminum foil, and a G-26 needle is used to position the mouse. Autoclave the surgical instruments before the experiment at 121°C for 20 min.

2. Prepare and sterilize the surgical tools needed for sample collection (Figure 1).

On the day of sacrifice

3. Set the temperature of the water bath with agitation at 37°C, 200 rpm.
4. Pre-chill the centrifuge to 4°C.
5. Prepare PDL complete medium.
  **CRITICAL:** Filter medium through a 0.22 μm polyether sulfone (PES) membrane before use.
  a. Equilibrate to room temperature 1 h before the experiment.
6. Prepare the PDL digestion buffer.
  **CRITICAL:** Prepare freshly before the experiment and filter the medium through a 0.22 μm polyether sulfone (PES) membrane before use.
  a. Equilibrate to room temperature 1 h before the experiment.

### Preparation to subculture and grow primary mouse PDLCs

**Timing: 1 h**

On the subculture date

7. Pre-chill the centrifuge to 4°C.
8. Prepare PDL complete media.
  **CRITICAL:** Filter the medium through a 0.22 μm polyether sulfone (PES) membrane before use.
  a. Equilibrate to room temperature 1 h before the experiment.
9. Thaw and equilibrate the ReagentPack Subculture Reagents (store at −20°C) to room temperature before the experiment for 1 h.

***Optional:*** Thaw ReagentPack Subculture Reagents at 4°C 2 days before subculture day to minimize the time to equilibrate the reagents to room temperature.

### Preparation for the osteogenic differentiation assay using primary mouse PDLCs

**Timing: 1 h**

10. Prepare osteogenic differentiation medium (ODM).
  **CRITICAL:** Filter the medium through a 0.22 μm polyether sulfone (PES) membrane before use.
  a. Equilibrate to room temperature 1 h before the experiment.
    ***Note:*** It is recommended to prepare ODM weekly to ensure the differentiation results.

### Preparation for collecting primary mouse bone marrow cells

**Timing: 2 h (for step 11)**

**Timing: 20 min (for steps 12 and 13)**

The day prior to the sacrifice

11. Sterilize the instruments needed to collect samples.

On day of sacrifice

12. Pre-chill the centrifuge to 4°C.
13. Prepare the osteoclast supplemental medium.
  **CRITICAL:** Filter the medium through a 0.22 μm polyether sulfone (PES) membrane before use.
  a. Equilibrate to room temperature 30 min before the experiment.

### Preparation for the osteoclastogenesis assay using primary mouse bone marrow cells

**Timing: 15 min**

14. Prepare the osteoclast differentiation medium.
  **CRITICAL:** Filter the medium through a 0.22 μm polyether sulfone (PES) membrane before use.
  a. Equilibrate to room temperature for 30 min before the experiment.

## Key resources table

**Table:** 

REAGENT or RESOURCE	SOURCE	IDENTIFIER
**Chemicals, peptides, and recombinant proteins**		

Liberase solution	Merck	Cat# 05401119001
SCBM stromal cell BulletKit	Lonza	Cat# CC-3205
SCBM stromal cell growth basal medium	Lonza	Cat# CC-3204
SCGM stromal cell growth medium SingleQuots supplements and growth factors	Lonza	Cat# CC-4181
ReagentPack subculture reagents	Lonza	Cat# CC-5034
Alpha-MEM	Gibco	Cat# 11900-024
Ascorbic acid	Wako	Cat# 013-19641
Β-glycerophosphate	Sigma	Cat# G9422-10G; CAS# 154804-51-0
GlutaMAX supplement	Life Technologies	Cat# 35050061
Penicillin-streptomycin solution (×100)	FUJIFILM Wako	Cat# 168-23191
Dexamethasone	Sigma	Cat# D8893; CAS# 50-02-2
Alizarin red S	FUJIFILM Wako	Cat# 100375; CAS# 130-22-3
ACK lysing buffer	Gibco	Cat# A1049201
Recombinant mouse M-CSF	R&D	Cat# 416-ML-010
Recombinant mouse TRANCE/RANK L/TNFSF11 protein	R&D	Cat# 462-TR-010
Fetal bovine serum Netherlands origin	Serana Europe GmbH	Cat# S-FBS-NL-015
DPBS	FUJIFILM Wako	Cat# 043-29791
DPBS, no calcium, no magnesium	Gibco	Cat# 14190136
UltraPure DNase/RNase-free distilled water	Invitrogen	Cat# 10977015

**Critical commercial assays**		

Alkaline phosphatase (ALP) staining kit	Cosmo Bio	Cat# PMC-AK20
Tartrate-resistant acid phosphatase (TRAP) staining kit	Cosmo Bio	Cat# PMC-AK04F-COS

**Experimental models: Organisms/strains**		

C57BL/6 mice, male or female, 4–72 weeks	Charles River Laboratories (Kanagawa, Japan)	RRID:MGI:2159769

**Software and algorithms**		

ZEN Blue imaging software	ZEISS	RRID:SCR_013672
ImageJ software	Schneider et al.^7^	RRID:SCR_003070
GraphPad Prism	GraphPad Software	RRID:SCR_002798

**Other**		

Dumont forceps	Multiple vendors	N/A
Suture-tying forceps	Multiple vendors	N/A
Forceps Perry 13 cm, curved forceps	Multiple vendors	N/A
Surgical scissors	Multiple vendors	N/A
Scalpel blades no. 10	Multiple vendors	N/A
Flexible-arm dissection light	Multiple vendors	N/A
Syringe 1 mL	Multiple vendors	N/A
Needle (20G and 26G)	Multiple vendors	N/A
Styrene foam 30 cm × 20 cm.	Multiple vendors	N/A
BioCoat collagen I 12-well clear flat bottom TC-treated multiwell plate, with lid	Corning	Cat# 354500
STEMFULL centrifuge tube	STEMFULL	Cat# MS-90150
EVOS M5000 microscope	Thermo Fisher Scientific	Cat# AMF5000
Leica EZ4 stereo microscope	Leica Microsystems	www.leica-microsystems.com
ZEISS Axio Imager 2 microscope	ZEISS	RRID:SCR_018876
HS all-in-one fluorescence microscope	KEYENCE	Cat# BZ-9000E

## Materials and equipment

**Table:** PDL digestion buffer

Reagent	Final concentration	Amount
FBS	2%	0.06 mL
Liberase (Merck, 05401119001)	2 Wünsch units/mL	0.46 mL (from stock concentration 13 Wünsch units/mL)
PBS	N/A	2.48 mL
**Total**	**N/A**	**3 mL**

Always prepare fresh and store at 4°C before the experiment.

### PDL complete medium

Use SCGM Stromal Cell Bullet Kit, which consists of the SCBM Stromal Cell Growth Basal Medium and the SCGM Stromal Cell Growth Medium SingleQuots Supplements and Growth Factors.

**Table:** 

Reagent	Final concentration	Amount
SCBM, Stromal Cell Basal Medium	N/A	500 mL
hFGF-B	N/A	0.5 mL
Insulin	N/A	0.5 mL
GA-1000	N/A	0.5 mL
FBS	5%	25 mL
**Total**	**N/A**	**526.5 mL**

Always prepare fresh before each assay. Store in dark at 4°C for up to 1 month.

**Table:** Osteogenic differentiation medium (ODM)

Reagent	Final concentration	Amount
Alpha-MEM	80%	386.25 mL
Ascorbic Acid (stock 20 mg/mL)	50 μg/mL	1.25 mL
Β-glycerophosphate	5 mM	2.5 mL
GlutaMAX Supplement	1%	5 mL
Penicillin/Streptomycin	1%	5 mL
FBS	20%	100 mL
**Total**	**N/A**	**500 mL**

Always prepare fresh before each assay. Store at 4°C for up to 1 month

**Table:** Osteogenic mineralization medium (OMM)

Reagent	Final concentration	Amount
Alpha-MEM	80%	386.25 mL
Ascorbic Acid (Stock 20 mg/mL)	50 μg/mL	1.25 mL
Β-glycerophosphate	5 mM	2.5 mL
GlutaMAX Supplement	1%	5 mL
Penicillin/Streptomycin	1%	5 mL
FBS	20%	100 mL
Dexamethasone (stock 5 mM)	10 nM	0.001 mL
**Total**	**N/A**	**500 mL**

Always prepare fresh before each assay. Store at 4°C for up to 1 month

***Alternatives:*** Use the prepared ODM and add Dexamethasone (final as 10 nM).

**Table:** Osteoclast supplemental medium

Reagent	Final concentration	Amount
Alpha-MEM	90%	9 mL
Recombinant Mouse M-CSF (stock 100 μg/mL)	5 ng/mL	0.5 μL
FBS	10%	1 mL
**Total**	**N/A**	**10 mL**

Always prepare fresh before each assay and store at 4°C before the experiment

**Table:** Osteoclast differentiation medium

Reagent	Final concentration	Amount
Alpha-MEM	90%	8.98 mL
Recombinant Mouse M-CSF (stock 100 u = μg/mL)	100 ng/mL	10 μL
Recombinant Mouse TRANCE/RANK L/TNFSF11 Protein (stock 100 μg/mL)	100 ng/mL	10 μL
FBS	10%	1 mL
**Total**	**N/A**	**10 mL**

Always prepare fresh before each assay and store at 4°C before the experiment.

## Step-by-step method details

### Primary mouse PDLCs collection

**Timing: 1–2 h**

Here, we describe steps to collect and culture primary mouse PDL cells before the experiment.

1. Euthanize the mice one by one and obtain the molars immediately after euthanasia.

***Optional:*** After euthanization by isoflurane, perform transcardiac perfusion with PBS and 4% paraformaldehyde before separating the maxilla and mandible.

**Figure 3:**
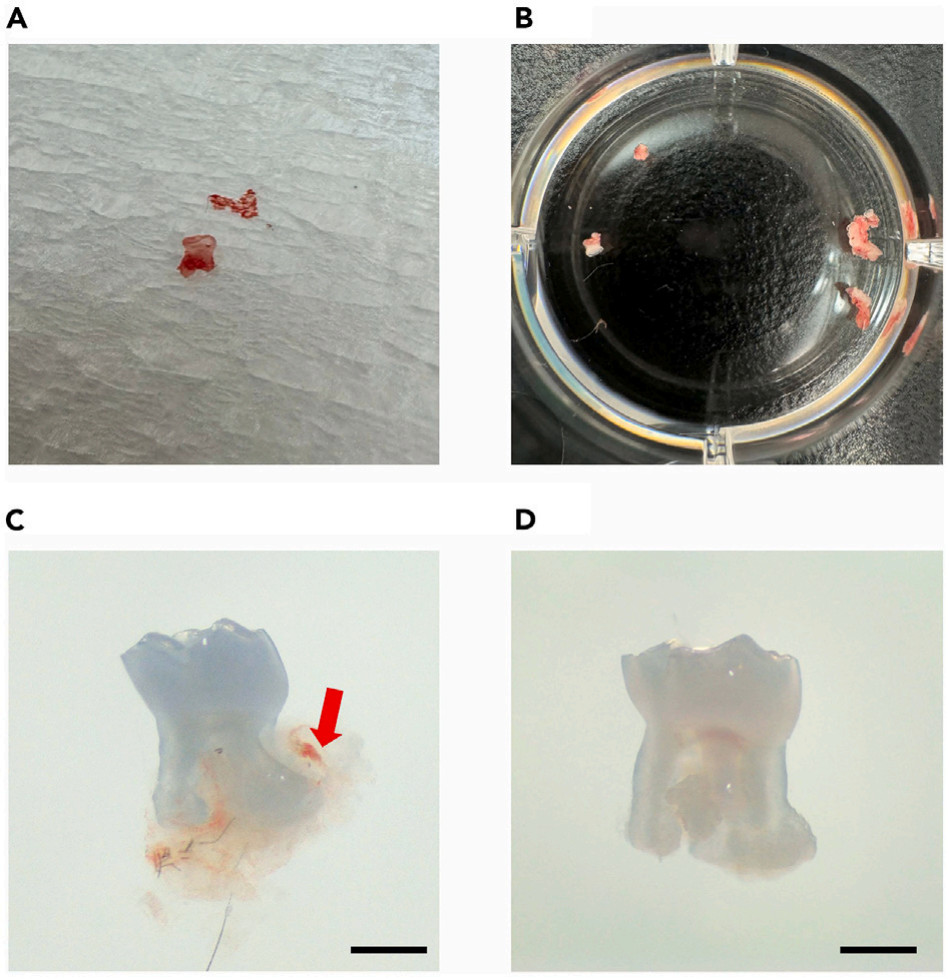
The molar after incubation in the PDL digestion buffer (A) The extracted maxillary second molar. (B) Incubate the extracted molars in PBS. (C and D) The extracted maxillary second molar under the Leica EZ4 stereo microscope. (C) The extracted molar with attached PDL tissues on the root surface (red arrow). (D) The extracted molar after incubation in the PDL digestion buffer. No PDL tissue is observed after enzyme digestion. Scale bar: 0.5 mm.

2. Obtain maxillary molars from the mice (Figure 2).
  a. Separate the maxilla and mandible.
    **CRITICAL:** It is important to carefully separate a maxilla from the mouse head so as not to break the alveolar bone around the maxillary molars.
  b. Carefully peel away and remove gingival tissue.
  c. Extract maxillary molars from the alveolar bone.
    ***Note:*** It is recommended to perform this step under a Leica S6 stereomicroscope or under a high-magnification field.
    **CRITICAL:** Do not forcefully extract tooth until the tooth is slightly mobile to avoid root fracture inside the alveolar bone.
  d. Collect extracted teeth in 1× PBS (with Ca^2+^ and Mg^2+^) in a 12-well plate.
  e. Flush the teeth three times with a syringe containing 1× PBS and transfer them to a new well containing PBS.
    **CRITICAL:** Do not let the teeth stay in PBS longer than 1 h as it will decrease the % of vital cells.
  f. Check under the microscope to confirm that the PDL tissue is attached to the root surface (Figure 3).
    ***Note:*** Upon visual inspection, the PDL tissues appear as a soft tissue exhibiting a light pink hue. This connective tissue intimately adheres to the tooth root surface and harbors a network of blood vessels.

**Figure 2:**
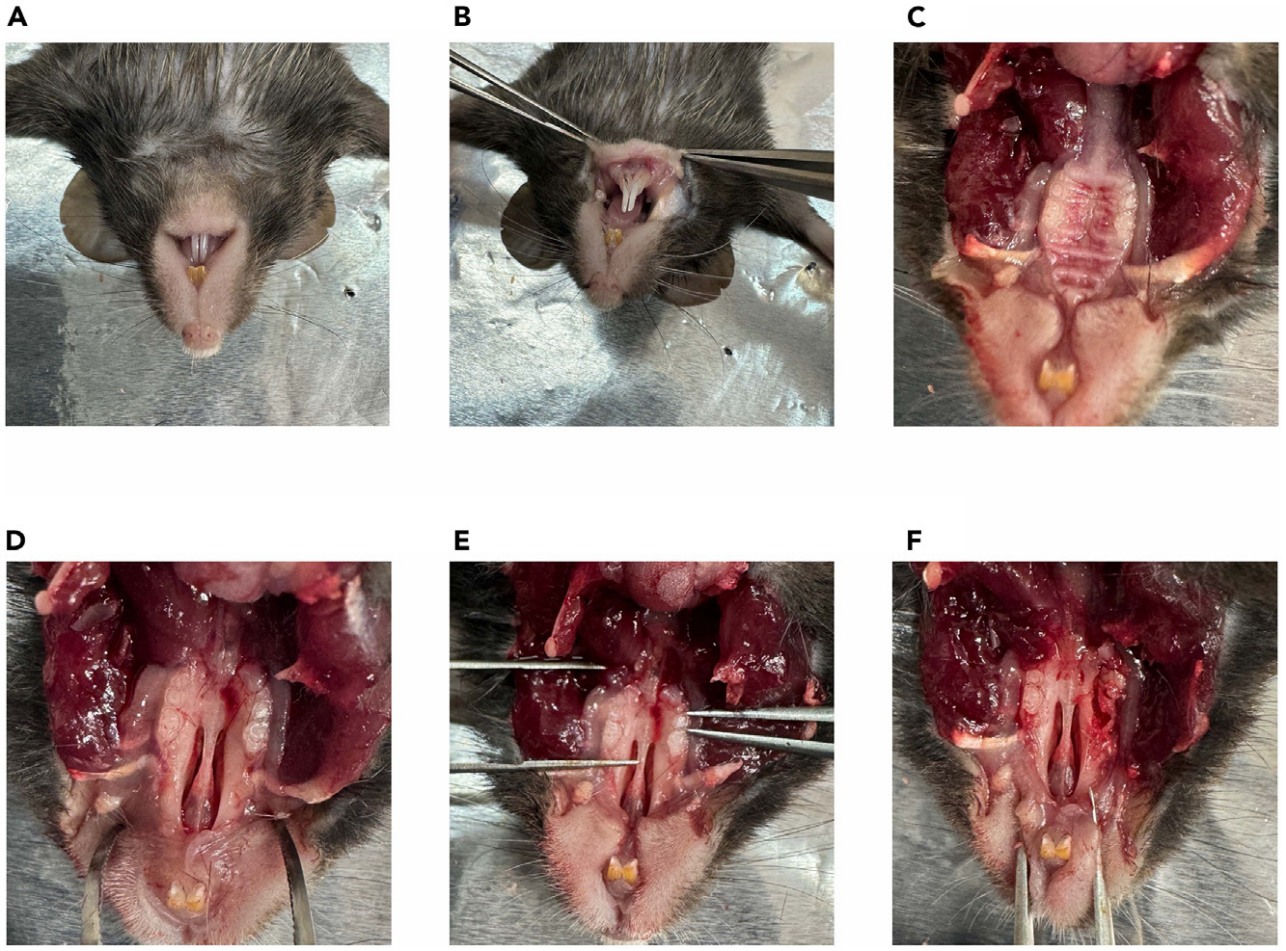
Surgical procedure to extract mouse maxillary molars (A) Position the mouse in a supine posture with its head oriented towards the operator. (B) Incise both sides of the cheek to facilitate access to the maxillary molars. (C) The optimal access for initiating the molar extraction process. (D) Remove the palatal gingiva and soft tissue surrounding the molars. (E) Tooth extraction positioning: On the left side, use an instrument to stabilize the maxilla; on the right side, use Dumont forceps to luxate the tooth and extract it from the tooth socket. (F) Extracted sockets following the removal of maxillary left molars.
3. Incubate the teeth in 1.5 mL of PDL digestion buffer per 15 mL low-binding tube (STEMFULL Centrifuge Tube).
  ***Note:*** STEMFULL is a centrifuge tube coated with an ultra-hydrophilic polymer that reduces cell loss by preventing nonspecific adherence to the tube walls. This coating ensures optimal cell recovery post-centrifugation.^8^
  a. Place the tubes in the incubator with agitation at 200 rpm for 20 min at 37°C.
  b. Add 15 μL of 0.5 M EDTA to each tube.
  c. Place the tubes in the incubator with agitation at 200 rpm for 10 min at 37°C.
  d. Add 9 mL of washing buffer that is equilibrated to room temperature (PBS containing 2% FBS).
  e. Centrifuge at 400 × *g* for 8 min at 4°C.
4. Resuspend the cell pellet in 2 mL of washing buffer.
  a. Filter the supernatant through a 70-μm cell strainer (BMS, BC-AMS-17001).
  b. Centrifuge at 320 × *g* for 5 min at 4°C.
5. Resuspend the cell pellet in 1 mL of washing buffer.
  a. Count the number of living cells.
    ***Note:*** The average number of live cells from a single second molar is 4 × 10^3^ cells.
  b. Add PDL complete medium to adjust the cell concentration at 10^4^ cells per mL.
6. Seed the cell in a culture vessel.
  a. Seed the cells at 10^4^ cells per well in a 12-well plate.
    **CRITICAL:** Use collagen-coated plates to increase cell attachment.
  b. Add 1 mL of cell suspension per well for a 12-well plate.
  c. Culture at 37°C in 5% CO_2_.
  d. Change the PDL complete medium every two days.
    ***Note 1:*** The average time to reach 80% confluent is one week.
    ***Note 2:*** Discard the cell flask after 8 weeks of culturing as PDLCs are not differentiated.
7. Subculture when cells reach 80% confluent to increase cell number for the actual experiment.
  a. Follow the manufacturer’s protocol. https://bioscience.lonza.com/lonza_bs/CH/en/download/product/asset/30937.
  b. Subculture from 12-well plate to T25 flask to increase cell number.

### Osteogenic differentiation from primary mouse PDLCs

**Timing: up to 28 days**

In this section, we describe steps to differentiate primary mouse PDL cells to osteoblast cells.

8. Obtain a confluent plate or flask of primary PDLCs. The representative images of primary mouse PDLC cultures are shown in Figure 4.
  ***Note:*** Below is the protocol for a T25 (25 cm^2^) flask culture.
  a. Aspirate the culture medium from the culture vessel.
  b. Rinse the cells once with 5 mL pre-warmed HEPES-BSS.
    **CRITICAL:** Skipping this step will lead to the failure of the trypsinization process to dissociate the attached cells, as the media contains proteins and calcium that neutralize the trypsin.^9^
  c. Aspirate the HEPES-BSS from the flask.
  d. Add 2 mL of 0.25% Trypsin/EDTA solution.
  e. Allow the trypsin to cover the cell layer and incubate for 5 min at 37°C with CO_2_.
  f. Check the cell layer in the microscope.
    ***Note:*** 90% of the cells should be detached at this time and appear circular in shape, floating in the solution.
  g. Gently tap the culture vessel against the palm of the hand to accelerate the cell dissociation from the vessel.
    **CRITICAL:** If only a small number of cells detach, it is possible that the cells were not trypsinized for a sufficient duration. Wait for 30 s and tap again. Should detachment not occur, extend the wait not longer than 10 min and continue tapping every 30 s thereafter.
  h. Neutralize the trypsin in the culture vessel with 4 mL of room temperature Trypsin Neutralizing Solution.
  i. Transfer the detached cells to a sterile 15 mL centrifuge tube.
    ***Optional:*** A sterile 15 mL low-binding tube can be used to obtain a higher cell recovery.
    i. Examine the empty flask under the microscope to confirm the harvest was successful.
    ii. The remaining cells should be less than 5%.
  j. Centrifuge the harvested cells at 220 × *g* for 5 min at 4°C.
    i. Aspirate the supernatant.
    ii. Add 1 mL of the PDL complete medium.
    iii. Slowly resuspend the cell pellet with a micropipette.

**Figure 4:**
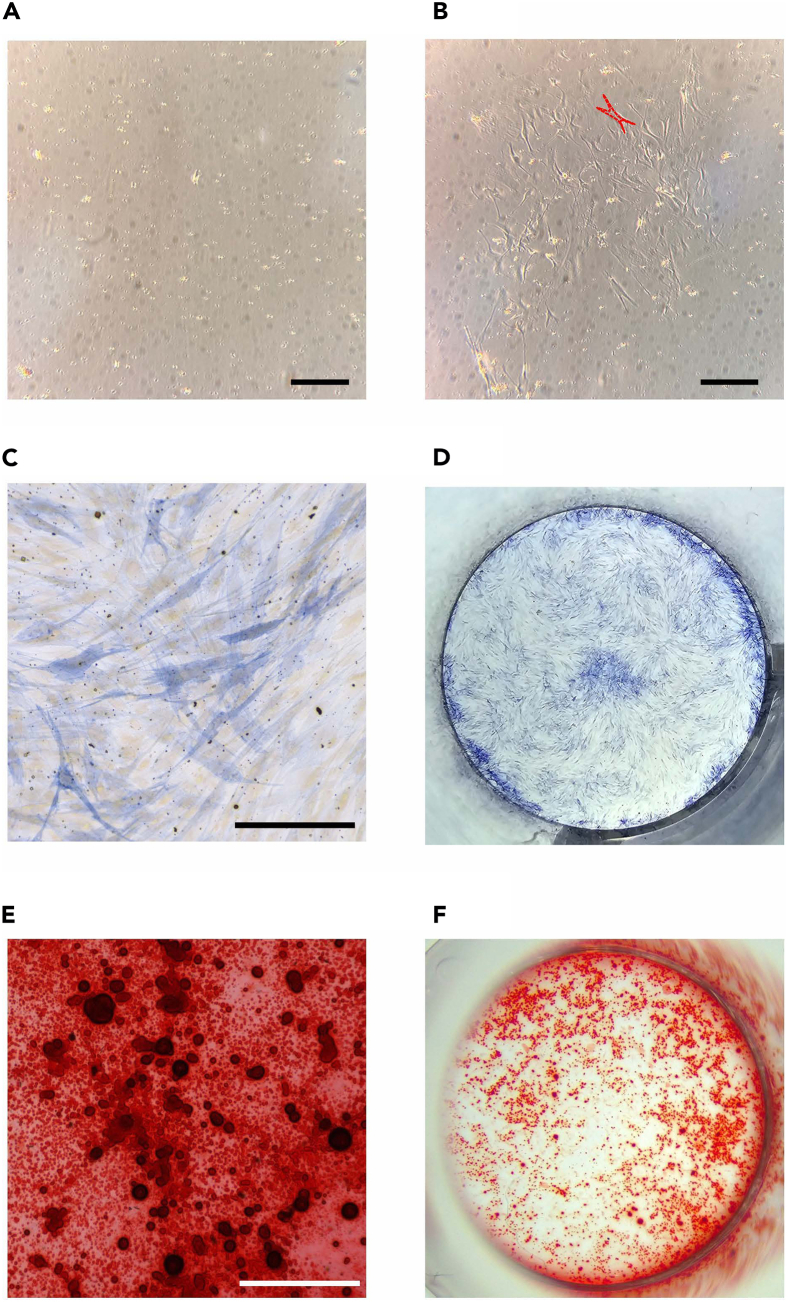
Osteogenic differentiation of primary mouse PDLCs (A) Primary PDLCs at Day 2 after collection. The attached PDLCs predominantly maintain a circular shape. (B) Primary PDLCs at Day 6 after collection. The majority of cells exhibit a spindle-shaped morphology (fibroblast-like cells, red dots). (C–F) Differentiated cells from PDLCs at Day 28 of the osteogenic differentiation assay. (C, D) Cell cultures were stained with ALP stain. (C) Microscopic image showing ALP-positive cells from the cell cultures. (D) The entire well of cultured cells after ALP staining. Used for calculating the percentage of ALP-positive area. (E, F) Cell cultures were stained with ARS. (E) Microscopic image showing mineralized nodules visualized by ARS staining. (F) The entire well of the cultured cells after ARS staining. Used for calculating the percentage of the mineralization area. Scale bar: 300 μm.
9. Seed the primary mouse PDLCs into a cell culture plate.
  a. Calculate and adjust the live cell concentration to 2 × 10^4^ cells per cm^2^ in the desired volume for the experiment.^10^
    ***Note:*** The working volume for each well of a 48-well plate is 200 μL.
  b. Add the adjusted cell suspension to the wells.
  c. Culture in the incubator for two days at 37°C with 5% CO_2_.
    ***Note:*** Replace the PDL complete medium every two days and continue culture if the cells do not reach 80% confluency.
10. Culture the primary mouse PDLCS in the osteogenic differentiation medium.
  a. Aspirate the old medium from the culture plate.
  b. Replace with the osteogenic differentiation medium (ODM).
  c. Replace the medium every two days.
  d. Continue culture for 21 days.^11^
11. Culture the primary mouse PDLCS in the osteogenic mineralization medium.
  a. Aspirate the old medium from the culture plate.
  b. Wash the cell culture wells with 1× PBS (with Ca^2+^ and Mg^2+^) once.
  c. Replace with the osteogenic mineralization medium (OMM).
  d. Replace the medium every two days.
  e. Continue culture for up to 28 days.

**CRITICAL:** Prolonged culture in the OMM will lead to abundant calcification, or calcification nodules are greater than 80% of culture area, in the cell culture wells. This causes difficulty to compare the calcification between experimental groups as the calcification area will be used to calculate the percentage of calcification area to represent the properties of each experimental agent to induce osteogenic differentiation.

12. Stain the cells for an analysis of osteogenic differentiation assay.
  a. Stain cells with Alizarin Red S (ARS) to visualize the calcification nodules from the differentiated osteoblasts (step-by-step explanation in the next protocol).
  b. Stain cells using the Alkaline Phosphatase (ALP) staining kit (Cosmo Bio) to visualize alkaline phosphatase activity from the differentiated osteoblasts. The protocol for ALP staining follows the manufacturer’s protocol. https://search.cosmobio.co.jp/cosmo_search_p/search_gate2/docs/PMC_/AK20.20230828.pdf
    i. Obtain the histological images after ALP staining (Figure 4).
      ***Note:*** Take histological images after the staining solution has been removed and replaced with DPBS solution.
    ii. Obtain stereological images to calculate the percentage of the ALP-positive area (Figure 4).
      **CRITICAL:** Air dry at room temperature overnight before capturing images.
      ***Note:*** In the case of well plates larger than 48 wells, it is imperative to utilize a compact camera for capturing images of an entire well. This is essential because the minimum magnification of the Leica EZ4 Stereo Microscope is insufficient to encompass the entirety of a well, making it impractical for calculating the percentage of calcification based on ALP staining.
    iii. Analyze the ALP-positive area using ImageJ software.^7^
    iv. Compare the ALP-positive area to the control group using Prism software.

### Alizarin Red S (ARS) staining

**Timing: 2 h**

Here, we describe steps to stain cell cultures with Alizarin Red S staining after undergoing osteogenic differentiation for 28 days.

***Note:*** Below is the protocol for a 48-well plate.

Adjust the volume depending on the type of culture plate.

13. Prepare 2% Alizarin Red S solution in ddH_2_O and adjust the pH to 6.3–6.4.
  a. Dissolve 1 g Alizarin Red S in 50 mL of ddH_2_O.
  b. Vortex for 30 s.
  c. Prepare 0.2 M ammonium hydroxide (NH_4_OH) in ddH_2_O.
  d. Adjust pH of Alizarin Red S solution to 6.3–6.4 with NH_4_OH solution.^12^^,^^13^
  e. Filter the solution through a 0.22 μm membrane.

***Note:*** Store at 4°C in protected from light up to one month.

14. Aspirate the osteogenic differentiation medium from the culture plate.
15. Add 0.2 mL DPBS (no calcium, no magnesium) to each well to wash the cells.
16. Fix the cells with 0.2 mL 10% formalin.
  a. Incubate for 1 h at room temperature.
  b. Remove formalin with a pipette.
    **CRITICAL:** Do not touch the bottom of the well as this will lead to the detachment of the cell layer.
  c. Wash the cells with 0.2 mL of ddH_2_O twice.
17. Add 0.2 mL of freshly made Alizarin Red S Solution (pH 6.3–6.4).
  a. Incubate in the dark for 45 min at room temperature.
18. Aspirate Alizarin Red S solution.
  a. Wash the cells twice with 0.2 mL ddH_2_O.
19. Obtain histological images after ARS.
  a. Carefully aspirate the wash buffer from each well.
  b. Add 0.2 mL DPBS (no calcium, no magnesium) per well.
    ***Note:*** Take histological images after the DPBS solution has been added.
  c. Examine the wells via a microscope and take photos using a stereo microscope (Figure 4).
20. Obtain stereological images to calculate the percentage of calcification using ARS.
  a. Aspirate remaining liquid.
  b. Dry overnight at room temperature.
  c. Take photos using a Leica EZ4 Stereo Microscope (Figure 4).
    ***Note:*** In the case of well plates larger than 48 wells, it is imperative to utilize a compact camera for capturing images of an entire well. This is essential because the minimum magnification of the Leica EZ4 Stereo Microscope is insufficient to encompass the entirety of a well, making it impractical for calculating the percentage of calcification based on ARS staining.
  d. Analyze the ARS-positive area using ImageJ software.^7^
  e. Compare the ARS-positive area to the control group using Prism software.

### Primary mouse bone marrow cells collection

**Timing: 6 h**

In this section, we describe steps to collect primary mouse bone marrow cells to obtain macrophages as the cell progenitors for osteoclasts.

***Note:*** Below is the protocol for 96-well plate.

21. Euthanize mice by isoflurane or cervical dislocation.
22. Collect femurs (Figure 5).
  a. Make a cut in the skin near the thigh.
  b. Separate the skin layers to find the femoral head.
  c. Remove the muscle around the femur.
  d. Rotate the femur around an axis to dissociate from a socket.
  e. Remove the remaining soft tissue.
  f. Cut both ends of the femur to make a cylinder appearance.
  g. In a petri dish, inject MEM-alpha medium, which is equilibrated to room temperature, to wash the femur by syringe.
    ***Note:*** Use 2 mL of MEM-alpha medium per one femur.
  h. Collect the fluid and transfer through a 40-μm cell strainer.
  i. Centrifuge at 400 × *g* for 10 min at 4°C.
  j. Tilt the tube to discard the supernatant and aspirate any remaining supernatant.

**Figure 5:**
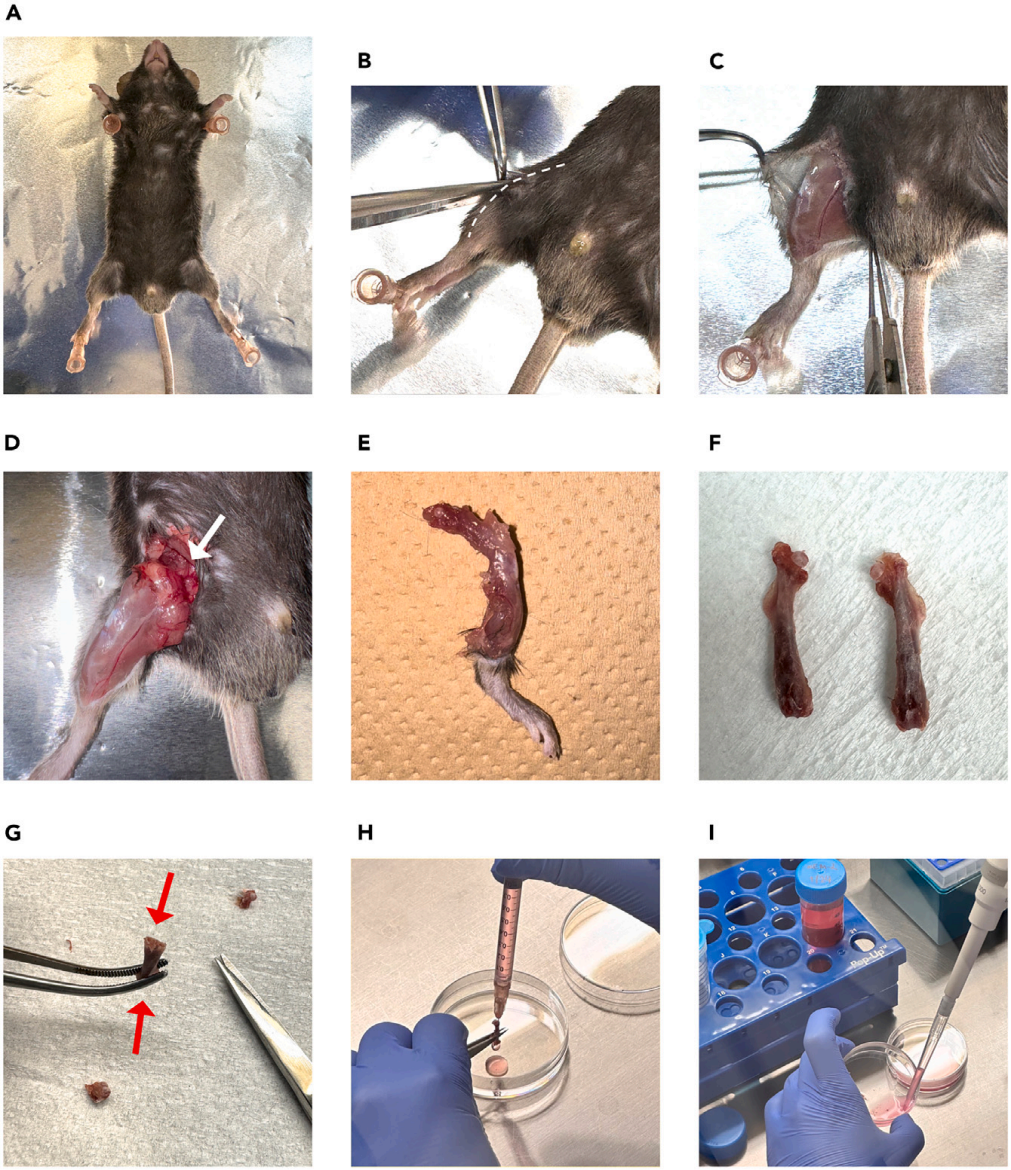
Surgical procedure to collect primary mouse bone marrow cells (A) Position the mouse in a supine posture with its head oriented away from the operator. (B) Grasp the skin at the leg and cut along the leg (white dashed line). (C) Retract the skin to expose the muscle layer. (D) Evert the skin and cut the muscles to locate the femoral head. Rotate the femur around an axis to aid in finding the femoral head (white arrow) and dislocate it from the socket. (E) Detached leg with remaining soft tissues. (F) Bilateral femurs after cleaning and removing soft tissues. (G) Cut both ends of the femur (red arrows) to create a cylindrical appearance for media flushing. (H) Use a syringe with a 26-G needle filled with MEM-alpha medium to flush the femur and collect the cells in a Petri dish. (I) Collect the cell suspension and proceed to the subsequent steps.
23. Add 2 mL of RBC lysis buffer.
  a. Gently resuspend the cell pellet by pipetting.
  b. Incubate in RBC lysis buffer for 8 min at room temperature.
    **CRITICAL:** Red blood cells will impede macrophage attachment to the bottom of the well if the incubation period is not long enough.
  c. Add 2 mL of MEM-alpha medium.
  d. Centrifuge at 400 × *g* for 10 min at 4°C.
  e. Tilt the tube to discard the supernatant and aspirate any remaining supernatant.
  f. Resuspend the cell pellet in 1 mL of MEM-alpha medium.
24. Perform cell counting and adjust the cell suspension as needed.

***Note 1:*** For non-coated 96-well plate, the recommended number of live bone marrow cells for seeding is 10^5^ cells per mL.^14^

***Note 2:*** The typical range of live cells from one femur is 2–4 × 10^6^ cells.

25. Add the osteoclast supplemental medium to bone marrow cell suspension to adjust the cell concentration.
26. Add 0.1 mL of the cell suspension per well (10^4^ cell per well) of 96-well plate.
27. Place the plate in the incubator for 3 h at 37°C in 5% CO_2_.

### Osteoclastogenesis assay using primary mouse bone marrow cells

**Timing: 6 days**

Here, we describe the procedures for inducing the differentiation of primary mouse bone marrow cells into osteoclasts, along with the subsequent analytical methodology involving TRAP staining conducted at the conclusion of the assay.

28. After 3 h, aspirate the osteoclast supplemental medium.
29. Wash with 1× PBS (with Ca^2+^ and Mg^2+^) once and replace with the osteoclast differentiation medium.
30. Culture for 3 days at 37°C in 5% CO_2_.
31. On the 3^rd^ day, check the cells under the microscope to confirm differentiation.

**Figure 6:**
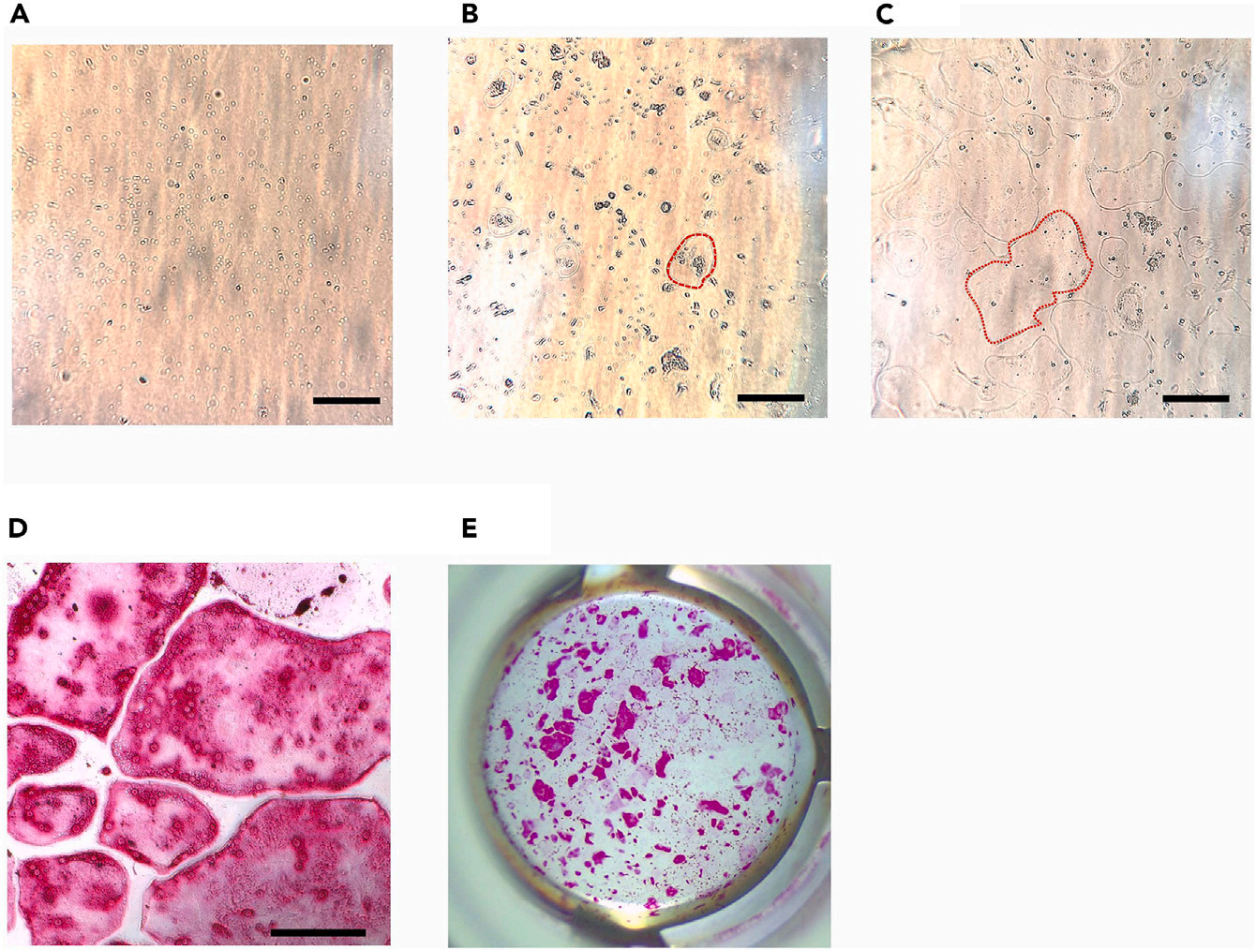
Procedure to collect primary mouse bone marrow cells (A) Primary bone marrow cells after 3 h incubation in osteoclast supplemental medium. The cells predominantly maintain a small circular shape. (B) Osteoclasts differentiated from primary bone marrow cells on Day 3 after collection. Multinucleated cells and cell fusion should be observed at this time point (red dots). (C) Osteoclasts differentiated from primary bone marrow cells on Day 6. Giant multinucleated cells were observed (red dots) before staining with a TRAP staining kit. (D and E) Cell cultures after staining with a TRAP staining kit. (D) Microscopic image showing giant multinucleated cells in cell culture. These multinucleated cells manifest a cytoplasmic stain in hues of red or purple when treated with reagents from the TRAP staining kit, indicative of the TRAP enzymatic activity. (E) The entire well of mouse bone marrow cells after TRAP staining. Used for calculating the percentage of the TRAP-positive area. Scale bar: 300 μm.

***Note:*** Small multinucleated cells and/or cell fusion should be observed (Figure 6).

32. Aspirate the old medium and replace it with the osteoclast differentiation medium.
33. Continue culture for up to 7 days at 37°C in 5% CO_2_.
34. On the 6^th^ day, check under the microscope to confirm differentiation.

***Note:*** Large multinucleated cells should be observed (Figure 6).

35. Stain the cells using the TRAP staining kit (Cosmo Bio) following the manufacturer’s protocol. https://search.cosmobio.co.jp/cosmo_search_p/search_gate2/docs/PMC_/AK04F.20231019.pdf.
36. Obtain histological images after TRAP staining (Figure 6).
  a. Carefully aspirate the ddH_2_O wash buffer.
  b. Add 0.1 mL DPBS (no calcium, no magnesium) per well.
    ***Note:*** Take histological images after adding the DPBS solution.
  c. Examine via a microscope and take photos using an EVOS M5000 microscope.
37. Obtain stereological images to calculate the percentage of the TRAP-positive area (Figure 6).
  a. Aspirate the remaining liquid.
  b. Dry overnight at room temperature.
  c. Take photos using a Leica EZ4 Stereo Microscope.
    ***Note:*** In the case of well plates larger than 48 wells, it is imperative to utilize a compact camera for capturing images of an entire well. This is essential because the minimum magnification of the Leica EZ4 Stereo Microscope is insufficient to encompass the entirety of a well, making it impractical for calculating the percentage of calcification based on ARS staining.
  d. Analyze the TRAP-positive area using ImageJ software.^7^
  e. Compare the TRAP-positive area to the control group using Prism software.

## Expected outcomes

### Primary mouse PDLCs

For six maxillary molars from one mouse, the total number of PDLCs should range 1–10 × 10^5^ cells. PDLCs can be combined from several mice and centrifuged prior to cell counting. The viability of each sample should be over 60%. Representative images of PDLCs in culture are shown in Figure 4.

Starting the molars collection from 3 mice, the live cells of collected PDLCs are suggested to seed on a single well of 12-well plate to expand the cells until it is confluent. PDLCs cultured in a 12-well plate should be 80% confluent within one week. Cells should be subcultured from a single 12-well plate to T25 flask to expand the cell number. A T25 flask should be confluent within one week after subculture from one well of a 12-well plate. One confluent flask of T25 flask should range 1–2 × 10^6^ cells and can be used for two plates of the 48-well plate. For 48-well plate, the appropriate number of cells for seeding is 5 × 10^3^ cells/well.

### Characterization of mineralization and differentiated osteoblasts from primary mouse PDLCs after osteogenic differentiation

Anticipated outcomes from *in vitro* experiments focusing on osteoblast differentiation from primary mouse PDLCs involve several key observations. The outcomes should include the formation of osteoblasts, characterized by their ability to produce mineralized extracellular matrix and their enzymatic activity of alkaline phosphatase.

In this experiment, the formation of mineralized nodules detected by ARS staining is key to successful osteoblast differentiation. The anticipated outcome of ARS staining in the context of osteoblast differentiation involves the visualization and quantification of mineralized matrix formation, demonstrating complete osteogenic differentiation. Alizarin red is a dye that binds to calcium ions, enabling the detection of mineralized nodules formed by osteoblasts. The staining results in the formation of a distinctive red color in areas where calcium deposits have accumulated, reflecting the presence of a mineralized extracellular matrix produced by differentiated osteoblasts.^15^ This outcome is critical for assessing the effectiveness of osteoblast differentiation protocols and for studying factors influencing bone mineralization. The representative images of the calcification nodules after the osteogenic differentiation are shown in Figure 4.

ALP staining is a widely utilized method for evaluating osteogenic differentiation. The anticipated result of alkaline phosphatase staining in osteoblasts entails visualizing enzymatic activity, particularly alkaline phosphatase activity that is important enzyme for bone mineralization. This staining produces a colored substrate at sites exhibiting alkaline phosphatase activity within the osteoblasts. Alkaline phosphatase’s role in the mineralization process involves hydrolyzing inorganic pyrophosphate, a mineralization inhibitor, emphasizing its importance in bone development. Detection and activity of alkaline phosphatase in osteoblasts indicates the generation of a mineralized extracellular matrix.^16^ The expected outcome of ALP staining is the identification of stained areas in osteoblasts, confirming their differentiation and active participation in bone mineralization. The representative images of ALP-positive cells after osteogenic differentiation are shown in Figure 4.

### Characterization of differentiated osteoclasts from primary mouse bone marrow cells

When using primary mouse bone marrow cells, common findings, and observations in osteoclastogenesis experiments should include the formation of giant multinucleated cells or the generation of osteoclasts with multiple nuclei. The formation of giant multinucleated cells is a key outcome in successful osteoclastogenesis, as these cells are generally characterized by their substantial, multinucleated nature. Moreover, successful experiments should show evidence of cell fusion during the differentiation process, as osteoclastogenesis involves the fusion of precursor cells to form multinucleated osteoclasts. The representative images of osteoclast differentiation are shown in Figure 6.

TRAP staining is used to characterize osteoclasts via the activity of tartrate-resistant acid phosphatase. When treated with staining reagents, osteoclasts manifest a cytoplasmic stain in hues of red or purple, indicating the enzymatic activity associated with TRAP. This particular method of staining shows the activity of the TRAP enzyme, which is active under acidic conditions, facilitating the localization of osteoclasts in close proximity to sites of bone resorption.^17^ The resulting staining outcome not only aids in qualitatively assessing the distribution of osteoclasts but also provides valuable insights into TRAP-related enzymatic activity linked with bone remodeling. Furthermore, TRAP staining offers the quantitative data that holds significance in studies focused on bone metabolism. The representative images of TRAP-positive cells are shown in Figure 6.

## Limitations

When collecting PDLCs from multiple donors, variability may exist between batches of harvested cells, resulting in varying degrees of growth efficiency. After starting cell cultures using diverse sets of collected PDLCs, only those wells showing optimal growth characteristics are retained for prolonged cultivation and subsequently subjected to the osteogenic differentiation assay. If starting with six maxillary molars derived from a single mouse, it is expected that only enough PDLCs will be collected for one well of a 12-well plate to reach 80% confluency within one week.

Due to the extended duration of PDLC culture, spanning a requisite period of two months to achieve the necessary cell density and osteoblast differentiation, it is imperative to exercise meticulous care in handling the cell cultures. It is essential to maintain sterile conditions and vigilantly monitor the cultures for any indications of contamination. In the event that contamination is observed in any well, the optimal course of action is to discard the entire plate. This precautionary measure aims to curb the proliferation of contaminants and prevent the preservation of contaminated cells, which could potentially give rise to secondary contamination in subsequent processes.

In addition, cellular heterogeneity poses a significant challenge in the culture of primary mouse macrophages in bone marrow cells, underscoring the importance of establishing rigorous control over *in vitro* conditions to thoroughly delineate macrophage responses to differentiate into osteoclasts.^18^

In summary, the existing protocols provide guidelines for sample collection, cell culture techniques, and staining procedures to visualize data for one of the parameters analyzed in the successful differentiation assay. Nevertheless, it is strongly advised to conduct molecular biology assays to validate the gene and protein expression of specific cell markers following cell differentiation.

## Troubleshooting

### Problem 1

It is difficult to extract mouse maxillary molars, especially the first maxillary molar. (step 2 of the primary PDLCs collection).

### Potential solution

Introduce a 20-gauge needle between the first and second molars to facilitate tooth loosening.^9^ For the first molar, use the tip of Dumont forceps to establish a gap between the tooth and alveolar bone. Then, firmly hold the tooth and gently luxate it. Upon achieving mobility, securely grasp the tooth and extract it. Caution should be exercised, as mishandling may result in tooth flip, necessitating the removal of any contaminated tooth from the collected samples.

### Problem 2

The number of collected PDLCs is low and not sufficient for cell culture (step 5 of the primary PDLCs collection).

### Potential solution

Generally, one mouse, from which six maxillary molars can be collected, is sufficient for the PDLCs to be seeded in a single well of a 12-well plate. Use periodontal microsurgery instruments or surgical blade for scraping of PDL tissues from the root surfaces.^8^^,^^19^ Moreover, careful attention to timing is essential during the collection of PDLCs, an operation exceeding 1 h has been observed to lead to a cell viability below 50%.

### Problem 3

The PDLCs detach from the culture vessel during culture and/or experiment (step 7 and 10 of the primary PDLC culture and differentiation).

### Potential solution

Use a collagen-coated plate or flask to ensure proper attachment of PDLCs to the surface during cell culture. Exercise caution when aspirating media from the cell culture to avoid touching the bottom of the well, as this could disrupt the cell layer and result in PDLC detachment. When adding medium to a well, position the pipette tip at the side wall and slowly depress the pipette plunger to gently introduce the medium into the well.

### Problem 4

The mineralized nodules are not observed in cell culture after ARS staining. (step 18 of ARS staining after osteogenic differentiation).

### Potential solution

Use the PDLCs for osteogenic differentiation or supplementary experiments within two months post-collection. Extended culture of PDLCs may result in the attenuation of their osteoblastic differentiation potential. Additionally, it is imperative to prepare fresh ODM and OMM weekly to maintain the nutrients required for cell growth during the assay. The appearance of calcification nodules, typically manifested as white spots, is commonly observed around the second week of osteogenic differentiation.

### Problem 5

The multinucleated cells are not observed on the 3^rd^ day of the assay. (step 31 of the primary mouse bone marrow cells collection).

### Potential solution

Check the quality of bone marrow cells collected during collection process. Exercise caution not to over-incubate the cell pellet in the ACK lysis buffer, as this may lead to a decrease in cell viability. Nevertheless, insufficient incubation can result in inadequate red blood cell lysis, diminishing osteoclastogenesis due to interreference from RBCs present in the cell culture.^14^ It is essential to prepare fresh osteoclast-supplemental and osteoclast differentiation media for each experiment, prior to media changes, and to equilibrate the media to 37°C before adding it to the cells. Additionally, appropriate reconstitution and proper storage of crucial reagents, such as M-CSF or recombinant RANK-L, are imperative to maintain protein function and ensure osteoclastogenesis results.

## Resource availability

### Lead contact

Further information and requests for resources and reagents should be directed to and will be fulfilled by the lead contact, Tomoki Maekawa (Maekawa-t@dent.niigata-u.ac.jp).

### Technical contact

Technical questions on executing this protocol should be directed to and will be answered by the technical contact, Kridtapat Sirisereephap (Kridtapat.s@chula.ac.th).

### Materials availability

• Cell culture plates generated in this study belong to the collections of The Center for Advanced Oral Science, Graduate School of Medical and Dental Sciences, Niigata University and can be studied upon request.

### Data and code availability

• All data reported in this paper will be shared by the lead contact upon request.
• This paper does not report original code.
• Any additional information required to reanalyze the data reported in this work paper is available from the lead contact upon request.
